# pH‐Dependent Protonation of Surface Carboxylate Groups in PsbO Enables Local Buffering and Triggers Structural Changes

**DOI:** 10.1002/cbic.201900739

**Published:** 2020-03-05

**Authors:** Lisa Gerland, Daniel Friedrich, Linus Hopf, Eavan J. Donovan, Arndt Wallmann, Natalja Erdmann, Anne Diehl, Martin Bommer, Krzysztof Buzar, Mohamed Ibrahim, Peter Schmieder, Holger Dobbek, Athina Zouni, Ana‐Nicoleta Bondar, Holger Dau, Hartmut Oschkinat

**Affiliations:** ^1^ Leibniz-Forschungsinstitut für Molekulare Pharmakologie Department of NMR-Supported Structural Biology Robert-Rössle-Strasse 10 13125 Berlin Germany; ^2^ Freie Universität Berlin Department of Biology, Chemistry and Pharmacy Thielallee 63 14195 Berlin Germany; ^3^ Max-Delbrück-Centrum für Molekulare Medizin Robert-Rössle-Strasse 10 13125 Berlin Germany; ^4^ Freie Universität Berlin Department of Physics, Theoretical Molecular Biophysics Arnimallee 14 14195 Berlin Germany; ^5^ Humboldt-Universität zu Berlin Institute of Biology Philippstrasse 13 10099 Berlin Germany; ^6^ Freie Universität Berlin Department of Physics, Biophysics and Photosynthesis Arnimallee 14 14195 Berlin Germany

**Keywords:** NMR spectroscopy, pH titration, photosystem II, p*K*_a_ values, protonation

## Abstract

Photosystem II (PSII) catalyzes the splitting of water, releasing protons and dioxygen. Its highly conserved subunit PsbO extends from the oxygen‐evolving center (OEC) into the thylakoid lumen and stabilizes the catalytic Mn_4_CaO_5_ cluster. The high degree of conservation of accessible negatively charged surface residues in PsbO suggests additional functions, as local pH buffer or by affecting the flow of protons. For this discussion, we provide an experimental basis, through the determination of p*K*_a_ values of water‐accessible aspartate and glutamate side‐chain carboxylate groups by means of NMR. Their distribution is strikingly uneven, with high p*K*_a_ values around 4.9 clustered on the luminal PsbO side and values below 3.5 on the side facing PSII. pH‐dependent changes in backbone chemical shifts in the area of the lumen‐exposed loops are observed, indicating conformational changes. In conclusion, we present a site‐specific analysis of carboxylate group proton affinities in PsbO, providing a basis for further understanding of proton transport in photosynthesis.

## Graphical abstract

**Over a barrel**: We determined the p*K*_a_ values of surface glutamic and aspartic acid residues in isolated PsbO, a Photosystem II subunit, by means of NMR‐monitored pH titration experiments. Groups of p*K*_a_ values around 3.5 and 5 were clustered on opposite sides of the β‐barrel.

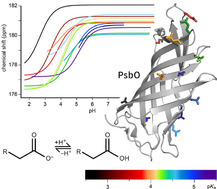

## Introduction

Photosynthesis involves a light‐dependent primary reaction, effecting the splitting of water, and a light‐independent secondary reaction, during which carbon dioxide is assimilated.^1^ The primary reaction is mediated by Photosystems I (PSI) and II (PSII), linked by a chain of redox‐active molecules.

PSII, located in the thylakoid membranes of photosynthetically active cells, splits two water molecules into four protons, four electrons, and one dioxygen molecule at the oxygen‐evolving center (OEC) containing a Mn_4_CaO_5_ cluster.^2^, ^3^ Because high proton concentrations in the vicinity of the OEC are counterproductive in this process, continuous removal of protons is required. The O_2_‐formation step of PSII is indeed inhibited at low pH, with a half‐inhibition pH of 4.6.^4^ At lower pH, there is most likely an irreversible inactivation of PSII caused (or initiated) by Ca^2+^ release. For these reasons, the photosynthetic organism will prevent a luminal pH as low as 4.6. A luminal pH above 5 is optimal for PSII function. In the thylakoid lumen, on the other hand, a pH close to 5 is needed for ensuring a trans‐thylakoid pH gradient that suffices for driving ATP synthesis. A mechanism avoiding lower luminal pH values is energy‐dependent quenching (qE), a component of the intensively studied non‐photochemical quenching (NPQ),^5^, ^6^, ^7^, ^8^ during which heat is generated instead of photochemical energy. The initiation of qE is directly coupled to the luminal pH and is the fastest component of NPQ.^9^, ^10^

**Figure 1:**
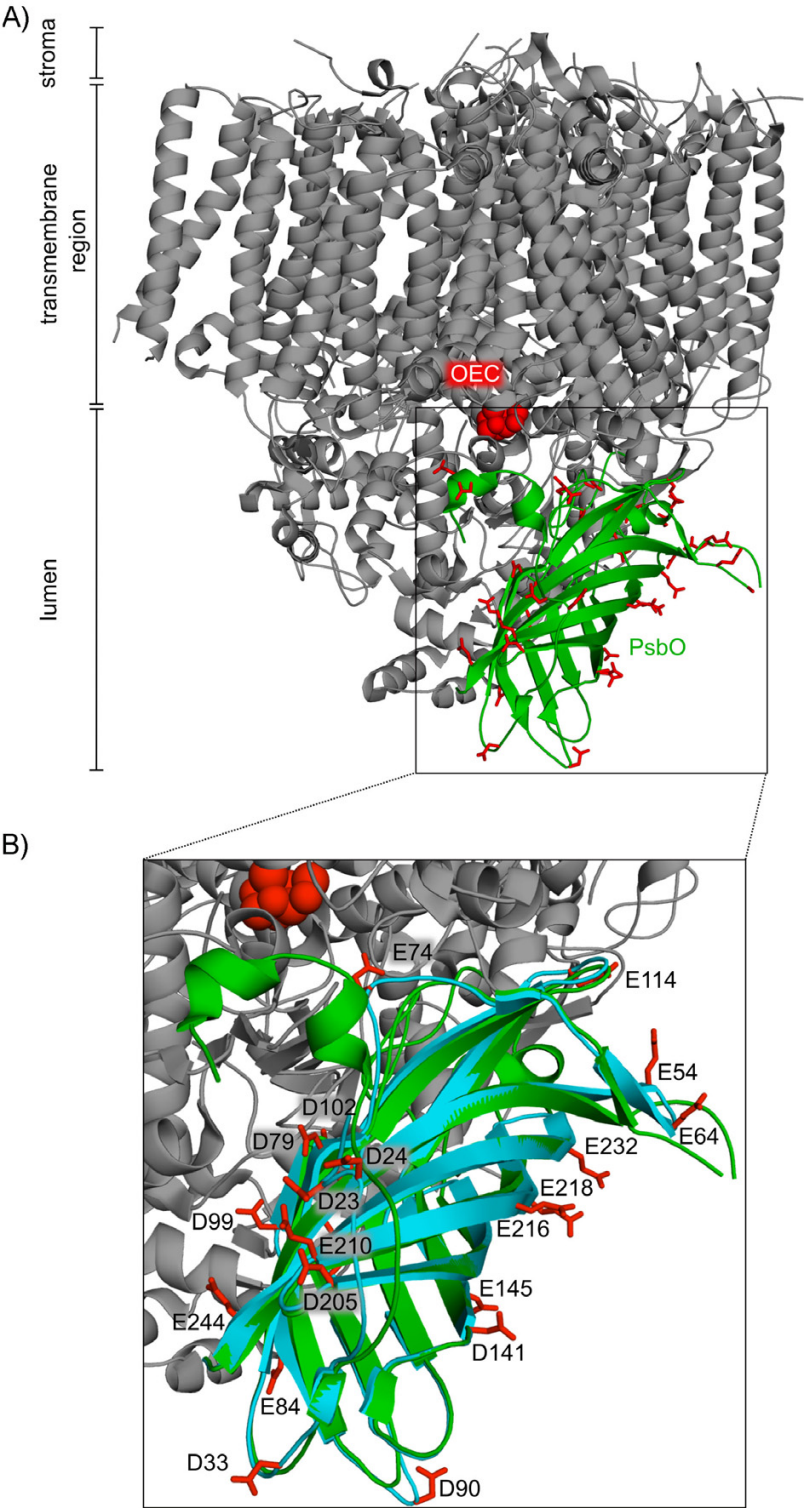
Location of PsbO in photosystem II monomer. A) The crystal structure of PSII of *Thermosynechococcus vulcanus* (PDB ID: 3WU2^22^) shows the localization of PsbO (green). It is thought to contribute to proton transport from the OEC (depicted in red spheres) to the thylakoid lumen. PsbO features a number of surface aspartate and glutamate carboxylate groups^[11, 12]^ (red). B) Overlay of full‐length PsbO in green and the shorter PsbO‐β in teal. Glutamate and aspartate residues of PsbO‐β are depicted as sticks, and the residue numbers are consistent with the numbering of full‐length PsbO.

It has been suggested that the evolutionarily conserved extrinsic PSII subunit PsbO, which is located at the luminal side of PSII (Figure 1 A), might provide a small buffering capacity^11^ or pathways for proton removal,^12^, ^13^, ^14^ or might act as a proton antenna,^11^ in addition to its established function in controlling the chloride and calcium concentrations at the OEC, thereby (and possibly also by other means) stabilizing the manganese complex.^14^, ^15^, ^16^ A pH buffer functionality of the PsbO carboxylate groups could transiently avoid acidification of the thylakoid lumen, for conditions of fluctuating light intensities (but not under continuous illumination), that easily and often occur in a natural habitat. This process might be relevant in a time segment of 5–20 s,^17^ which is a typical time regime for luminal acidification after an increase in light intensity in an intact organism. The fast luminal acidification is directly coupled to a protective quenching of an excited antenna state (qE), followed by activation of slower protection mechanisms.^5^, ^6^

X‐ray crystallography^2^, ^14^ and computation^13^, ^18^, ^19^ suggested an extended proton network around the OEC, serving as starting point for proton transport pathways towards the thylakoid lumen. PsbO interacts with subunits D1 and D2 of PSII through the loops Asp158–Lys188 and Asp222–Ala228,^20^ and their residues Asp158, Asp222–Asp224, His228, and Glu229 are part of a putative proton exit pathway.^12^, ^13^, ^14^, ^18^, ^21^ The smallest distance between PsbO and the OEC is 17 Å, as measured in a PSII crystal structure (PDB ID: 3WU2^22^). As shown in Figure 1, PsbO reaches out far into the luminal space. It exposes a considerable number of carboxylate groups on the surface of its β‐barrel.^12^, ^18^, ^20^ These are largely solvent‐accessible, show a surprisingly high degree of conservation^20^ (Figure S1), and might potentially affect proton flows from the OEC.^11^, ^12^, ^15^, ^18^, ^20^ Proton or water transport directly through the interior of the β‐barrel is unlikely because it is blocked by bulky hydrophobic residues.^23^

A tendency of PsbO to undergo pH‐dependent structural changes was initially mentioned in conjunction with an observed hysteresis in acid–base titration experiments of isolated PsbO.^24^ Through a combination of molecular simulations and crystal structure analyses, it has been observed that upon deprotonation of the carboxylate dyad—Glu97(residue 90 in investigated construct), Asp102(95)—the Asp102 side chain moves away from Glu97 and Lys123(116).^25^ This happens at pH values between 6 and 10. These amino acid residues are at the interface between PsbO, PsbU, and CP43. Recent computations and analyses of crystal structures of PSII indicated that the interface between PsbO and PsbU hosts several hydrogen‐bonded water molecules and an extensive network of water‐mediated bridges between carboxylate groups; the energy barrier for proton transfer from Asp102(95) is high, and this could be interpreted as suggesting that a proton bound at this site could remain on the surface of the protein, at least transiently.^26^ To provide further experimental data relating to PsbO's potential role in proton management, we determined the p*K*_a_ values of aspartate and glutamate side‐chain carboxylate groups on the surface of the PsbO barrel by NMR. For this purpose, we employed a soluble construct^25^ lacking the loops close to the OEC and studied the acidic residues directly situated on the barrel (Figure 1 B). In detail, the PsbO‐β construct used here and studied before by X‐ray crystallography^25^ did not contain the N‐terminal residues 1–15 or the residues in three loop regions (55–63, 149–192, and 220–231). The loop residues were omitted because they would not be structured in the isolated subunit lacking PSII interactions partners. All aspartate and glutamate residues remaining in the PsbO‐β construct are on the outer surface of full‐length PsbO within the PSII complex and solvent‐accessible. In the following sections, we employ the numbering of full‐length PsbO and indicate trimmed construct numbers in brackets.

Over the pH range of 2.0–7.0, the ^13^C chemical shifts of side‐chain carboxylate groups can change strongly,^27^ depending on the formation or disruption of a hydrogen bond. This provides the basis for determining the p*K*_a_ values of such moieties through pH titrations and fitting the course of chemical shift changes to the Henderson–Hasselbalch equation^28^, ^29^ (Figure S2). Our assignment of the side‐chain carboxylate group signals of PsbO thus enabled the determination of 21 p*K*_a_ values in a residue‐specific manner. As one striking result, we found a strong regional difference of p*K*_a_ values on the protein surface, suggesting an influence on charge distribution, local pH, buffering capacity, and proton flow.

## Results

### Determination of p*K*_a_ values of glutamate and aspartate carboxylate groups

**Figure 2:**
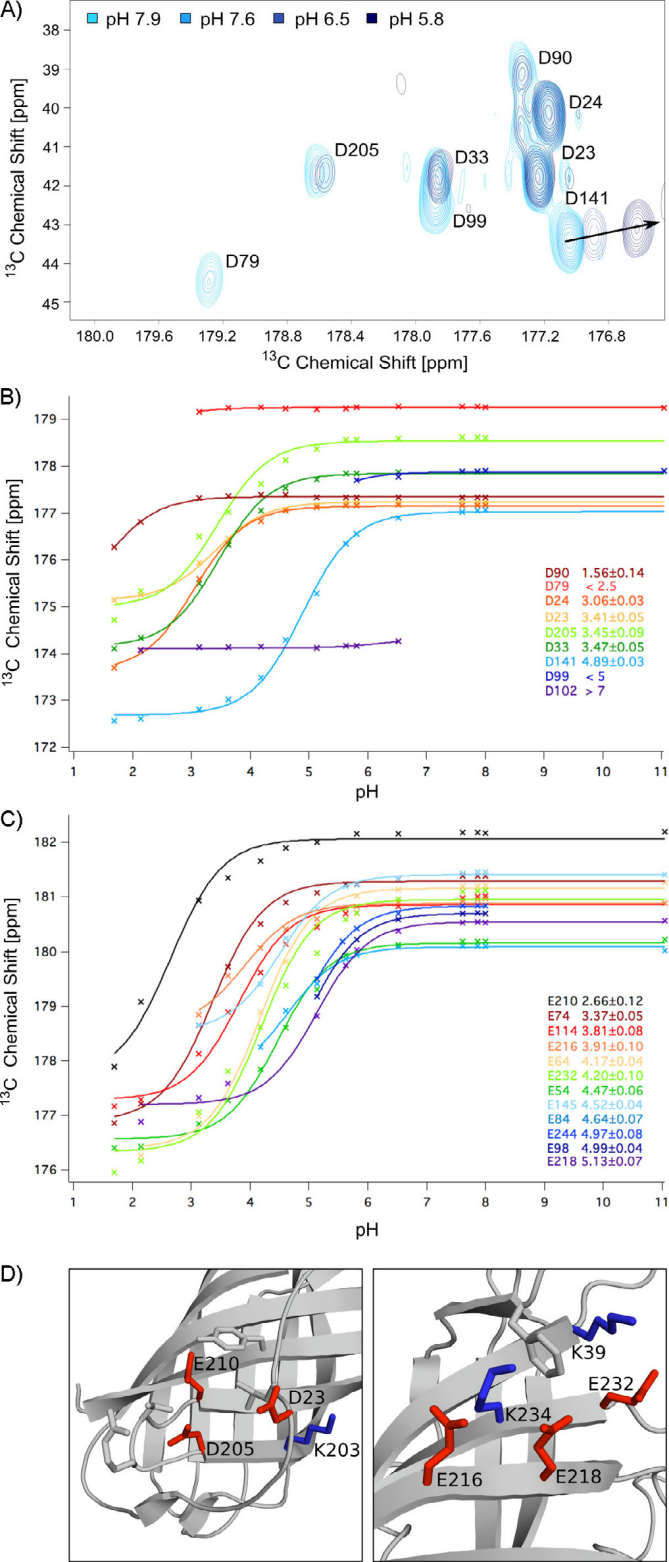
pH titration experiments and analysis. A) 2D CBCACO spectra at different pH values between pH 5.8 (dark blue) and pH 7.9 (light blue). The black arrow indicates the chemical shift changes of the Asp141 side‐chain carboxylate group carbon with the pH shift. B) Observed titration curves of aspartic acid residues and the obtained p*K*_a_ values (95 % confidence intervals are given as errors) are color‐coded from low (red) to high (blue) values. C) Titration curves of glutamic acid residues with p*K*_a_ values and 95 % confidence intervals are color‐coded from low (black) to high (purple). D) The side chain conformations of residues displaying side‐chain carboxylate group carbon resonances that do not shift in close agreement with the Henderson–Hasselbalch equation are shown in the crystal structure of PsbO‐β.^25^

We determined proton affinities of the glutamate and aspartate carboxylate groups by monitoring the chemical shifts of the ^13^C_γ_ and ^13^C_δ_ atoms, respectively. This required the assignment of the ^13^C_α_, ^13^C_β_, ^13^CO, ^15^N, and ^1^H^N^ resonances of PsbO‐β, which was achieved to 98 % completeness through triple‐resonance experiments (Figures S3 and S4). To assign the chemical shifts of side‐chain carbon atoms in glutamate and aspartate residues, we employed ^13^C‐detected, 3D CBCACO experiments.^30^ pH titrations were monitored by 2D CBCACO spectroscopy, yielding correlations involving ^13^C_γ_ and ^13^C_δ_ chemical shifts of aspartate and glutamate, respectively (Figure 2 A). We observed well‐resolved crosspeaks, thus enabling precise interpretation of chemical shift changes of the carboxylate group carbons upon pH titration; see, for example, Asp141(134). The corresponding crosspeaks display pH changes in the range of 7.6 to 5.8 as indicated by the black arrow. Using this approach, we determined the p*K*_a_ values for a one‐step proton‐exchange mechanism, applying a nonlinear least‐squares fit function. The titration curves of 21 aspartic and glutamic acid residues in PsbO‐β are shown in Figure 2 B and 2 C, respectively. In most cases, the applied Henderson–Hasselbalch equation fits the experimental data reasonably well, such as in the cases of residues Asp23(23) and Asp141(134). However, the fit is less perfect for Glu210(161), Glu218(169), Glu232(176), and Asp205(156). The titration curves of these residues deviate in areas adjacent to the inflection points, thus indicating proton exchange involving additional sites: that is, two‐step exchange mechanisms. Instructive examples are provided by the two clusters Asp23(23)/Glu210(161)/Asp205(156) and Glu216(167)/Glu218(169)/Glu232(176). In those clusters, protons are potentially shared through bifurcated hydrogen bonds (Figure 2 D, left and right), so deviations of several data points from the fit function (Figure S2 in the Supporting Information) are observed. The corresponding crosspeaks of Asp79(72) (Figure 2 B, red), Asp99(92) (Figure 2 B, dark blue), and Asp102(95) (Figure 2 B, violet) migrate during pH titration into a spectral region with heavy overlap of resonances, or vanish at certain pH values. For these reasons, some curves are incomplete and the p*K*_a_ value can therefore only be estimated as an upper or lower boundary.

Most of the glutamic acid p*K*_a_ values cluster around the standard value of 4.25 for glutamic acid in solution,^31^ with the two exceptions of Glu74(67) (p*K*_a_=3.37±0.05) and Glu210(161) (p*K*_a_=2.66±0.12) being more acidic.

The variance of the p*K*_a_ values of aspartic acid residues was strikingly high, with four of them differing strongly from the standard value of 3.67±0.04^31^ for aspartic acid in solution. Asp102(95) (p*K*_a_>7) and Asp141(134) (p*K*_a_=4.89±0.03) exhibit very high p*K*_a_ values, whereas Asp90(83) (p*K*_a_=1.56±0.14) is the most acidic. To our surprise, this amino acid is part of a flexible loop, which implies a higher p*K*_a_ for this residue. The low p*K*_a_ value of Asp79(72) (p*K*_a_<2.5) might be explained by a neighboring disulfide bridge, which might stabilize the negative charge, leading to a lower p*K*_a_ value.

In summary, we determined the p*K*_a_ values for 21 aspartate and glutamate carboxylate groups on the PsbO‐β surface. All are solvent‐accessible in full‐length PsbO within the PSII complex. The p*K*_a_ of Glu97(90) is missing because crosspeaks involving the C_δ_ could not be assigned.

Calculation of p*K*_a_ values with ProPka 3.0^32^ (for details see the Supporting Information, in particular Figure S5) showed a reasonable agreement between predicted and measured p*K*_a_ values (Table 1), with the greatest difference for Glu98(91) (Δ=1.03) followed by Glu210(161) (Δ=0.97). As expected, the calculated values for residues with extreme p*K*_a_ values diverged more strongly from the measured data than those with values closer to the average p*K*_a_.

**Table 1:** p*K*_a_ values of aspartic acid and glutamic acid residues.

Asp residue no.	p*K*_a_ value	Glu residue no.	p*K*_a_ value
23	3.41±0.05	54	4.47±0.06
24	3.06±0.03	64	4.17±0.04
33	3.47±0.05	74	3.37±0.05
79	<2.5	84	4.64±0.07
90	1.56±0.14	98	4.99±0.04
99	<5	114	3.81±0.08
102	>7	145	4.52±0.04
141	4.89±0.03	210	2.66±0.12
205	3.45±0.09	216	3.91±0.10
		218	5.13±0.07
		232	4.20±0.10
		244	4.97±0.08

### Chemical shift perturbations (CSPs) of PsbO backbone amide groups

**Figure 3:**
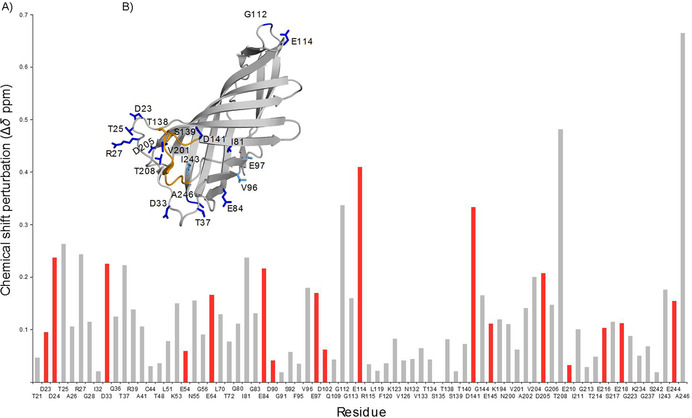
CSP measurements by 2D ^1^H,^15^N correlation spectroscopy to monitor conformation changes in PsbO‐β. A) ^15^N,^1^H CSPs of all residues that could be evaluated (acidic residues are highlighted in red, CSPs were calculated by a formula provided in the Experimental Section. The residues N55, G56, and G223 were inserted as loop replacements during trimming of the long construct and are not part of the original PsbO sequence. B) Residues with strongest CSPs plotted on the crystal structure of PsbO‐β (PDB ID: 5G39^25^), color‐coded from high (≥0.2, dark blue) to intermediate values (0.17≤CSP≤0.2, light blue). Residues with small CSPs (<0.17) are not displayed; amino acids that are part of the cyano loop are shown in orange and those involved in PSII dimer contacts as sticks.

In order to investigate whether pH changes over the range of 2–8 induce conformational rearrangements, and to establish whether or not the protein remains folded towards low pH, the titration was also monitored by 2D ^1^H,^15^N correlation spectroscopy. We observed strong chemical shift changes for several residues, together with negligible changes for the larger fraction of 2D crosspeaks (Figures 3 A, B and S6). Figure 3 A displays the pH‐dependent CSPs that we were able to analyze. In Figure 3 B the side chains of residues exhibiting the strongest shifts are shown within the PsbO‐β X‐ray structure, with indications of strength ranging from strong (>0.2) in dark blue to medium (0.17<CSP<0.2) in light blue. Small changes below 0.17 are not displayed because we consider them less significant. On the whole, the residues showing the largest changes are clustered towards the loops on the luminal side of the barrel, with Thr25(25), Thr208(159), and Ala246(191) showing the largest effects. Interestingly, the cyano loop that mediates contacts between PSII dimers^33^ is surrounded by residues with amide group signals that are pH‐sensitive; hence it is not excluded that conformational changes modulate the dimer contacts. We noticed only intermediate CSP values for residues of the proposed structural switch region: Glu98(91) and Asp99(92). Another residue of this switch region—Asp102(95)—shows an unexpectedly small CSP. Interestingly, we were only able to assign peaks for Asp102(95) from pH 3.1 to 6.5. The possibility of a moderate chemical shift change in this range agrees with Asp102(95) altering its position in the carboxylate dyad after deprotonation between pH 6 and 10.

## Discussion

**Figure 4:**
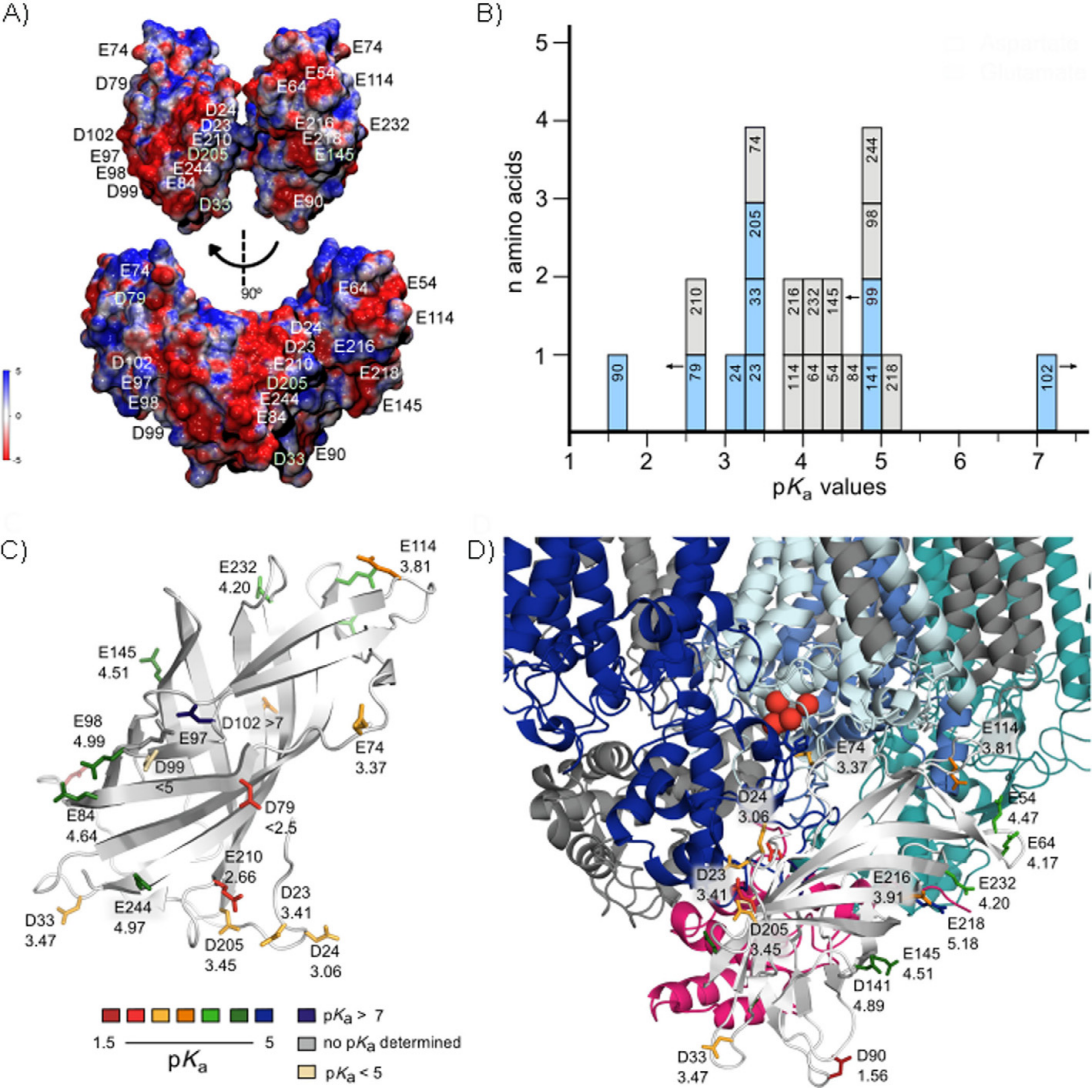
Distribution of p*K*_a_ values on the surface of PsbO‐β: interpretation in the context of PSII. A) Electrostatic potentials of amino acid residues plotted onto a surface created with van der Waals distances of PsbO*‐*β. B) Histogram demonstrating the p*K*_a_ distribution of carboxylic acid residues (p*K*_a_ axis divided into units of 0.25). Glutamic acid residues are shown in blue and aspartic acid residues in grey. The numbers indicate the amino acid position in full‐length PsbO. p*K*_a_ values of residues shown with an arrow could not be determined accurately; the arrows indicate that these values are below or above the given p*K*_a_. C) The crystal structure of the PsbO‐β construct of *T. elongatus* (PDB ID: 5G39^25^) shown in light gray, with glutamate and aspartate residues depicted as sticks, color‐coded according to their p*K*_a_ values. The residue numbers follow the nomenclature of full‐length PsbO. D) Close‐up of PsbO‐β (PDB ID: 5G38^25^) in complex with PSII (PDB ID: 2AXT^36^) and the associated proteins D1 (pale blue), D2 (blue), CP43 (dark blue), CP47 (turquoise), and PsbU (pink). The atoms of the OEC are shown as red spheres.

The PSII activity determines the luminal pH in thylakoids. Under optimal light conditions this can drop to a value of 5. Because a lower pH would strongly reduce the stability of PSII and inactivate the manganese complex, the activity of the photosystem needs to be regulated. The half‐inhibition point of the water oxidation reaction at PSII is already reached at pH 4.6.^4^ pH values below this point will lead to irreversible inactivation, believed to be controlled by calcium release.^34^ At pH values above 5, on the other hand, the transmembrane proton gradient (ΔpH) of the proton motive force (PMF) would be reduced and possibly too small to provide enough protons for ATP synthesis. This turns luminal pH control into an important task for photoactive plants and bacteria, and, due to its exposed position, PsbO might crucially contribute to this process. However, not all of the PsbO surface is solvent‐exposed (Figure 1 A and B). Apart from contacts with various PSII subunits, PsbO–PsbO contacts exist in the native‐like PSII X‐ray structure,^33^ formed by the cyano loops of two PsbO molecules (Figures 4 A and S7). Of the loop residues, Thr138(131) and Ser139(132) form hydrogen bonds to one another^33^ (Figure S7). The reported arrangement of dimers in a row is considered to be similar to the situation in native thylakoid membranes of spinach and pea.^35^

Prior to the interpretation of the p*K*_a_ values, and given a dimer arrangement of PSII, it is instructive to have a look at the electrostatic properties of the PsbO surface at pH 7. These are shown in Figure 4 A for the dimer as it exists in the crystal structure (PDB ID: 4PJ0^33^): as illustrated in the top panel, the contact areas between the two PsbO molecules are small, and a band of negative potential made up by residues Asp23(23), Asp24(24), Glu79(72), Glu205(156), and Glu210(161) is present on the other side, nearly forming a ring around the dimer. A second band of negative potential covers the area including Glu97(90), Glu98(91), Asp99(92), and Asp102(95). Apart from Asp99(92), all negatively charged residues are exposed to the lumen and are not involved in interactions with other PSII proteins (Figure S8). The p*K*_a_ values represent a quantification of pH effects on the charge of the protein. At first, it is surprising that the obtained values span a comparably wide region of the pH scale (Figure 4 B), ranging from 1.56 to 5.13 [potentially even higher for Asp99(92) and Asp102(95)], independent of the specific amino acid (Glu or Asp). For a detailed analysis, we show the side chains of the negatively charged residues with their p*K*_a_ values and color‐coded in the crystal structure of PsbO‐β (Figure 4 C, D). We observe a specific p*K*_a_ distribution with lower values clustering on the lumen‐exposed side of PsbO, whereas the area closer to PSII harbors residues with higher p*K*_a_ values, as revealed by analyzing the p*K*_a_ distribution on the PsbO‐β surface in complex with PSII (PDB ID: 2AXT^36^, Figure 4 D). A special role relating to buffering in the pH range between 4 and 7 is played by the carboxylate groups.^20^ Of the 22 side‐chain carboxylate groups, eight in our short PsbO‐β construct show p*K*_a_ values in the functionally relevant range from 4.3 to 5.1. Those residues are largely located at the side of PsbO facing the lumen (Figure 4 C, D).

Given the number of PsbO carboxylate groups relative to the extent of luminal space and protein density, PsbO's buffering capacity in this range is limited, yet those residues are in a suitable area: that is, not far from the potential proton release site (Figure 4 D). This might help in bypassing the first seconds of luminal acidification before downregulation of PSII activity through NPQ mechanisms takes over. The surprisingly wide distribution of p*K*_a_ values might affect the characteristics of PsbO's overall buffering capabilities. On generating the mean value of all curves, we find an elongated, less steep transition to the protonated state of the protein (Figure S9), with an inflection point around 4.1. Intriguingly, the p*K*_a_ values of the “negative band” involving residues Asp23(23), Asp24(24), Glu79(72), Glu205(156), and Glu210(161) are well or just below 4.0, which means they always remain deprotonated at any state of PSII activity. In contrast, the band of residues made up by Glu64, Glu84, Glu97, Glu98, Asp102, Asp141, Glu145, Glu218, Glu232, and Glu244 (Figure 4 C, D) become protonated over a pH range between 4.0 and 5.0. Because the low‐p*K*_a_ patch remains deprotonated down to a pH of 4.0, PsbO thus experiences a change in distribution of surface charges that might be the basis for a pH‐dependent switch.

In this context, it is interesting that pH‐dependent changes in the backbone chemical shifts indicative of structural changes are clustered at the luminal side of PsbO comprising the low‐p*K*_a_ patch (Figure 3 A, B). In particular, residues in the loops exposed to the lumen and in the corresponding ends of the β‐strands are affected, as well as a stretch involving Glu84(77), Val96(89), and Glu97(90). The structural changes extend towards the PsbU interaction area, so it is conceivable that they modulate the interaction between PsbO and PSII. A structural coupling between PsbO and OEC function has been concluded from FTIR difference spectroscopy.^15^, ^37^ Our data preclude pH‐induced opening of the barrel structure. The majority of residues in the upper part of the β‐barrel structure (Figure 3 B) feature small CSPs (<0.17) over a range from pH 3.1 to 7.9. Most of the protein thus shows high rigidity in our experiments, even at pH values below 4.

## Conclusions

We have determined, or estimated, 21 out of 22 p*K*_a_ values associated with aspartic acid and glutamic acid residues in PsbO‐β. Their uneven distribution on the luminal and PSII‐facing sides of the β‐barrel leads to an altered charge distribution at pH values between 3.5 and 5 within the thylakoids. This correlates with additional structural changes in PsbO as monitored by chemical shift changes of backbone amide groups. Overall, the induced structural changes might modify contacts between PsbO and other PSII proteins, as well as help to modulate the activity of the photosystem, thereby complementing the qE mechanism by a second route of pH‐induced downregulation of PSII activity. We note that upon protonation of crucial PsbO residues any related structural changes are likely to proceed on a very fast timescale, but because the luminal acidification (and thus PsbO side‐chain protonation) requires several seconds, the structural changes will become effective only on the seconds timescale. The rate‐determining step in the process is thus not these pH‐induced structural changes, but the acidification.

Further experiments based on our findings—for example, studying surface water molecules located at the interface between protein and surrounding fluid and that might play an important role in proton management—might prove informative. Room‐temperature neutron diffraction experiments for determining the locations of water molecules at the PsbO surface were performed by Bommer et al.^38^ Further work relating to surface water molecules could complement these studies of PsbO's role in PSII. Generating pH‐dependent PSII structures with X‐ray free electron lasers might elucidate the structural regulation of PsbO and other extrinsic subunits, as well as the interactions of PSII and PsbO with intrinsic subunits such as D1, D2, CP47, and CP43.

## Experimental Section

**Protein expression, purification, and sample preparation**: Details of the design and cloning of the *Thermosynechococcus elongatus* β‐barrel PsbO (PsbO‐β) construct are described in Bommer et al.^25^ The PsbO‐β construct in PET28a was transformed in *Escherichia coli* BL21(DE3) cells by following a standard heat shock protocol. Cells were plated and grown on lysogeny broth (LB) agar, with kanamycin (40 μg mL^−1^) at 37 °C overnight. Transformants were used to inoculate a LB preculture with kanamycin (25 °C, 180 rpm overnight). Cells were centrifuged for 10 min at 2000 *g* and RT, and the pellet was washed and resuspended in ^13^C and ^15^N M9 medium (2 L). Cells were grown at 37 °C and 170 rpm, reaching an OD of 0.4, at which expression was induced with isopropyl β‐d‐thiogalactopyranoside (IPTG, 1 mm), and cells were harvested after 5 h at 37 °C and 170 rpm.

Cell pellets (2 g) were resuspended in ice‐cold 2‐morpholin‐4‐ylethanesulfonic acid (MES) buffer (pH 6.6, 30 mm, 30 mL) with MgCl_2_ (2 mm). HS nuclease (250 units μL^−1^ Mobitec, Germany, 15 μL) was added, and cells were disrupted by using a microfluidizer (15 000 psi). Cell debris was spun down (30 min, 48 000 *g*, 25 °C), and the supernatant was incubated for 4 h at RT to digest the DNA effectively. Because the source of the PsbO is a thermophilic cyanobacterium we applied a purification protocol with an incubation step at 75 °C for 30 min. Most of the *E. coli* proteins denatured, with PsbO remaining in the supernatant after centrifugation (20 min, 48 000 *g*, 8 °C).

The supernatant was concentrated with Amicon Ultra 4 mL filters (3k MWCO) to 1.2 mL and added through a 0.45 μm filter. Size‐exclusion chromatography was performed (120 mL Superdex 75) with MES (pH 6.6, 30 mm). Fractions containing PsbO‐β were combined and concentrated 50‐fold to a final concentration of 35 mg mL^−1^ by using an Amicon Ultra 4 mL filter (3k MWCO).

For NMR titration measurements, the PsbO‐β sample concentration was adjusted to between 9 and 14 mg mL^−1^. To adjust the pH, samples were dialyzed overnight. A sodium phosphate buffer (20 mm) was used for a pH range between 5 and 8; for lower pH values phosphoric acid (20 %) was added, whereas for higher pH values NaOH (1 m) was used. All samples contained D_2_O (10 %); the pH and protein concentration were measured after the addition of D_2_O and before starting the NMR measurements.

**NMR spectroscopy**: Spectra were recorded with AV‐III‐600 spectrometers and cryogenically cooled probes (Bruker Biospin, Karlsruhe, Germany) at 300 K. The backbone was assigned by using HNCO, HNCACB, HN(CO)CACB, and HN(COCACB)CG 3D transverse relaxation‐optimized spectra (TROSY). The HNCO was recorded with an acquisition time of 51 ms in F_3_ (^1^H), 15.9 ms in F_2_ (^15^N), and 19.2 ms in F_1_ (^13^C), four scans, 512×48×48 complex points, and spectral widths of 10 000 (^1^H), 3012 (^15^N), and 2500 Hz (^13^C) in the direct and indirect dimensions. HNCACB, HN(CO)CACB, and HN(COCACB)CG were recorded with acquisition times of 51 ms in F_3_ (^1^H), 16.6 ms in F_2_ (^15^N), and 5.5 ms in F_1_ (^13^C), eight scans, 512×50×55 complex points, and spectral widths of 10 000 (^1^H), 3012 (^15^N), and 10 000 Hz (^13^C) in the direct and indirect dimensions. ^15^N,^1^H HSQC spectra were recorded with acquisition times of 51 ms in F_2_ (^1^H) and 85 ms in F_1_ (^15^N), eight scans, 512×256 complex points, and spectral widths of 10 000 and 3012 Hz in the direct and indirect dimension. The side‐chain assignment was performed with the aid of 3D CBCACO spectra. They were recorded with acquisition times of 51 ms in F_3_ (^13^C), 9.1 ms in F_2_ (^13^C), and 7 ms in F_1_ (^13^C), four scans, 512×64×70 complex points, and spectral widths of 10 000 (^1^H), 7042 (^15^N), and 10 000 Hz (^13^C) in the direct and indirect dimensions. 2D CBCACO titration spectra were recorded for 24 h with acquisition times of 51 ms in F_2_ (^13^C) and 6.4 ms in F_1_ (^13^C), 220 scans, 512×64 complex points, and spectral widths of 10 000 Hz in the direct and indirect dimensions. Assignment spectra were recorded with triply labeled protein (^2^H,^13^C,^15^N) whereas for the titration spectra and HSQC ^13^C/^15^N‐labeled PsbO was used. All spectra were processed by using Topspin 3.2 and the assignment was performed with CCPNmr Analysis 2.4.

**Calculation of CSP data**: CSPs were calculated by use of the following formula: [Eq. (1)]

(1) $$\mathrm{CSP}=\sqrt{\left[\Delta \delta \left(\mathrm{ppm}\right){}^{1}H\right]{}^{2}+\left[0.156\times \Delta \delta \left(\mathrm{ppm}\right){}^{15}N\right]{}^{2}}$$

Calculations are based on pH‐dependent shift differences [Δ*δ*(ppm)] observed in 2D ^15^N,^1^H HSQC spectra. We chose a pH range between 3.1 and 7.9 as standard for each residue if not stated otherwise. The data can be found in the Supporting Information.

**Constructing a model of PsbO–PsbO interactions in the PSII dimer of dimers**: Experiments have indicated that dimers of PSII can dimerize to tetramers,^33^, ^39^ and that in such dimers‐of‐dimers complexes, PsbO proteins mediate direct hydrogen bonds between the PSII dimers.^33^ We thus used the crystal structure PDB ID: 4PJ0 of the PSII dimer^40^ and the crystal structure PDB ID: 5G38 of PsbO‐β^20^ to generate a structural model of the PsbO‐mediated interactions between PSII dimers.

In the first step we used the transformation matrix reported with the crystal structure of the PSII dimer^40^ to generate a PSII dimer of dimers with PyMOL^41^ and we extracted the coordinates of the two PsbO copies that are in direct contact. In the second step we used visual molecular dynamics (VMD^42^) to overlap the structure of PsbO‐β with each of these two PsbO proteins. The overlap between PsbO‐β and full‐length PsbO was based on sequence analyses with ClustalW^43^ and on the findings of del Val et al.^20^

The electrostatic potential for PsbO‐β was calculated by using the PDB2PQR tool via the web interface^39^ to assign partial atomic charges from the CHARMM force field (Chemistry at Harvard Molecular Mechanics,^40^ and Adaptive Poisson–Boltzmann Solver (APBS)^41^ in VMD.

## Conflict of interest

*The authors declare no conflict of interest*.

## Supporting information

As a service to our authors and readers, this journal provides supporting information supplied by the authors. Such materials are peer reviewed and may be re‐organized for online delivery, but are not copy‐edited or typeset. Technical support issues arising from supporting information (other than missing files) should be addressed to the authors.

Supplementary

## References

[1] J. Barber , Philos. Trans. R. Soc. London Ser. B 2008, 363, 2665–2674. 18468983 10.1098/rstb.2008.0047 PMC2606770

[2] J. Kern , R. Chatterjee , I. D. Young , F. D. Fuller , L. Lassalle , M. Ibrahim , S. Gul , T. Fransson , A. S. Brewster , R. Alonso-Mori , et al., Nature 2018, 563, 421–425. 30405241 10.1038/s41586-018-0681-2 PMC6485242

[3] M. Suga , F. Akita , M. Sugahara , M. Kubo , Y. Nakajima , T. Nakane , K. Yamashita , Y. Umena , M. Nakabayashi , T. Yamane , et al., Nature 2017, 543, 131–135. 28219079 10.1038/nature21400

[4] I. Zaharieva , J. M. Wichmann , H. Dau , J. Biol. Chem. 2011, 286, 18222–18228. 21464129 10.1074/jbc.M111.237941 PMC3093894

[5] P. Müller , X. P. Li , K. K. Niyogi , Plant Physiol. 2001, 125, 1558–1566. 11299337 10.1104/pp.125.4.1558 PMC1539381

[6] A. V. Ruban , Plant Physiol. 2016, 170, 1903–1916. 26864015 10.1104/pp.15.01935 PMC4825125

[7] A. Kanazawa , E. Ostendorf , K. Kohzuma , D. Hoh , D. D. Strand , M. Sato-Cruz , L. Savage , J. A. Cruz , N. Fisher , J. E. Froehlich , D. M. Kramer , Front. Plant Sci. 2017, 8, 1–12. 28515738 10.3389/fpls.2017.00719 PMC5413553

[8] U. Armbruster , V. Correa Galvis , H. H. Kunz , D. D. Strand , Curr. Opin. Plant Biol. 2017, 37, 56–62. 28426975 10.1016/j.pbi.2017.03.012

[9] E. Kress , P. Jahns , Front. Plant Sci. 2017, 8, 1–17. 29276525 10.3389/fpls.2017.02094 PMC5727089

[10] A. Jajoo , M. Szabó , O. Zsiros , G. Garab , Biochim. Biophys. Acta Bioenerg. 2012, 1817, 1388–1391. 10.1016/j.bbabio.2012.01.002 22248669

[11] T. Shutova , V. V. Klimov , B. Andersson , G. Samuelsson , Biochim. Biophys. Acta Bioenerg. 2007, 1767, 434–440. 10.1016/j.bbabio.2007.01.020 17336919

[12] S. Lorch , S. Capponi , F. Pieront , A. N. Bondar , J. Phys. Chem. B 2015, 119, 12172–12181. 26334778 10.1021/acs.jpcb.5b06594

[13] F. Guerra , M. Siemers , C. Mielack , A. N. Bondar , J. Phys. Chem. B 2018, 122, 4625–4641. 29589763 10.1021/acs.jpcb.8b00649

[14] H. Ishikita , W. Saenger , B. Loll , J. Biesiadka , E. W. Knapp , Biochemistry 2006, 45, 2063–2071. 16475795 10.1021/bi051615h

[15] K. Ifuku , T. Noguchi , Front. Plant Sci. 2016, 7, 84. 26904056 10.3389/fpls.2016.00084 PMC4743485

[16] T. M. Bricker , J. L. Roose , R. D. Fagerlund , L. K. Frankel , J. J. Eaton-Rye , Biochim. Biophys. Acta Bioenerg. 2012, 1817, 121–142. 10.1016/j.bbabio.2011.07.006 21801710

[17] H. Dau , J. Photochem. Photobiol. B 1994, 26, 3–27.

[18] A. N. Bondar , H. Dau , Biochim. Biophys. Acta Bioenerg. 2012, 1817, 1177–1190. 10.1016/j.bbabio.2012.03.031 22503827

[19] D. Kaur , X. Cai , U. Khaniya , Y. Zhang , J. Mao , M. Mandal , M. R. Gunner , Inorganics 2019, 7, 14.

[20] C. del Val , A.-N. Bondar , Biochim. Biophys. Acta Bioenerg. 2017, 1858, 432–441. 28315679 10.1016/j.bbabio.2017.03.004

[21] J. Barber , K. Ferreira , K. Maghlaoui , S. Iwata , Phys. Chem. Chem. Phys. 2004, 6, 4737–4742.

[22] Y. Umena , K. Kawakami , J. R. Shen , N. Kamiya , Nature 2011, 473, 55–60. 21499260 10.1038/nature09913

[23] J. De Las Rivas , J. Barber , Photosynth. Res. 2004, 81, 329–343. 16034536 10.1023/B:PRES.0000036889.44048.e4

[24] T. Shutova , K. D. Irrgang , V. Shubin , V. V. Klimov , G. Renger , Biochemistry 1997, 36, 6350–6358. 9174350 10.1021/bi963115h

[25] M. Bommer , A. N. Bondar , A. Zouni , H. Dobbek , H. Dau , Biochemistry 2016, 55, 4626–4635. 27454911 10.1021/acs.biochem.6b00441

[26] L. Kemmler , M. Ibrahim , H. Dobbek , A. Zouni , A.-N. Bondar , Phys. Chem. Chem. Phys. 2019, 21, 25449–25466. 31713551 10.1039/c9cp03926k

[27] M. Tollinger , J. D. Forman-Kay , L. E. Kay , J. Am. Chem. Soc. 2002, 124, 5714–5717. 12010044 10.1021/ja020066p

[28] D. F. K. Julie , G. M. Clore , M. G. Angela , Biochemistry 1992, 31, 3442–3452. 1554726 10.1021/bi00128a019

[29] E. Bombarda , G. M. Ullmann , J. Phys. Chem. B 2010, 114, 1994–2003. 20088566 10.1021/jp908926w

[30] W. Bermel , I. Bertini , I. C. Felli , M. Piccioli , R. Pierattelli , Prog. Nucl. Magn. Reson. Spectrosc. 2006, 48, 25–45.

[31] R. L. Thurlkill , G. R. Grimsley , J. M. Scholtz , C. N. Pace , Protein Sci. 2006, 15, 1214–1218. 16597822 10.1110/ps.051840806 PMC2242523

[32] M. H. M. Olsson , C. R. Søndergaard , M. Rostkowski , J. H. Jensen , J. Chem. Theory Comput. 2011, 7, 525–537. 26596171 10.1021/ct100578z

[33] J. Hellmich , M. Bommer , A. Burkhardt , M. Ibrahim , J. Kern , A. Meents , F. Müh , H. Dobbek , A. Zouni , Structure 2014, 22, 1607–1615. 25438669 10.1016/j.str.2014.09.007

[34] A. Krieger , E. Weis , Photosynth. Res. 1993, 37, 117–130. 24317708 10.1007/BF02187470

[35] B. Daum , D. Nicastro , J. Austin , J. R. McIntosh , W. Kühlbrandt , Plant Cell 2010, 22, 1299–1312. 20388855 10.1105/tpc.109.071431 PMC2879734

[36] B. Loll , J. Kern , W. Saenger , A. Zouni , J. Biesiadka , Nature 2005, 438, 1040–1044. 16355230 10.1038/nature04224

[37] A. R. Offenbacher , B. C. Polander , B. A. Barry , J. Biol. Chem. 2013, 288, 29056–29068. 23940038 10.1074/jbc.M113.487561 PMC3790005

[38] M. Bommer , L. Coates , H. Dau , A. Zouni , H. Dobbek , Acta Crystallogr. Sect. F Biol. Commun. 2017, 73, 525–531. 10.1107/S2053230X17012171 PMC5619745 28876232

[39] T. J. Dolinsky , J. E. Nielsen , J. A. McCammon , N. A. Baker , Nucleic Acids Res. 2004, 32, W665–W667. 15215472 10.1093/nar/gkh381 PMC441519

[40] A. D. MacKerell, Jr. , D. Bashford , M. Bellott , R. L. Dunbrack , J. D. Evanseck , M. J. Field , S. Fischer , J. Gao , H. Guo , S. Ha , et al., J. Phys. Chem. B 1998, 102, 3586–3616. 24889800 10.1021/jp973084f

[41] N. A. Baker , D. Sept , S. Joseph , M. J. Holst , J. A. McCammon , Proc. Natl. Acad. Sci. USA 2001, 98, 10037–10041. 11517324 10.1073/pnas.181342398 PMC56910

[42] W. Humphrey , A. Dalke , K. Schulten , J. Mol. Graph. 1996, 14, 33–38. 8744570 10.1016/0263-7855(96)00018-5

[43] F. Madeira , Y. m. Park , J. Lee , N. Buso , T. Gur , N. Madhusoodanan , P. Basutkar , A. R. N. Tivey , S. C. Potter , R. D. Finn , R. Lopez , Nucleic Acids Res. 2019, 47, W636–W641. 30976793 10.1093/nar/gkz268 PMC6602479

