# Supplementary material for: pH‐Dependent Protonation of Surface Carboxylate Groups in PsbO Enables Local Buffering and Triggers Structural Changes
Source: Chembiochem. 2020 Mar 5;21(11):1597–604. doi: 10.1002/cbic.201900739 (PMC7318136; doi:10.1002/cbic.201900739)
Supplement: Supplementary file 1 — Supplementary [file CBIC-21-1597-s001.pdf]

CHEMBIOCHEM

Supporting Information

pH-Dependent Protonation of Surface Carboxylate Groups in PsbO Enables Local Buffering and Triggers Structural Changes

Lisa Gerland,*[a, b] Daniel Friedrich,[a, b] Linus Hopf,[a, b] Eavan J. Donovan,[a] Arndt Wallmann,[a] Natalja Erdmann,[a] Anne Diehl,[a] Martin Bommer,[c] Krzysztof Buzar,[d] Mohamed Ibrahim,[e] Peter Schmieder,[a] Holger Dobbek,[e] Athina Zouni,[e] Ana-Nicoleta Bondar,[d] Holger Dau,[f] and Hartmut Oschkinat*[a, b]

cbic_201900739_sm_miscellaneous_information.pdf

Table of Contents

Figure S1. Conservation pattern of PsbO surface carboxylates in plants and bacteria 3

Figure S2. Exemplary pH titration curve 4

Figure S3. ^{15}N-^{1}H HSQC spectrum of PsbO 4

Figure S4. Comparison of chemical shift index and crystal structure 5

Figure S5. Comparison of pK_a values with ProPka prediction program 6

Figure S6. ^{15}N-^{1}H HSQC titration spectra 6

Figure S7. PsbO-mediated interactions between Photosystem II (PSII) dimers 7

Figure S8. Interactions of PsbO with different subunits in a PSII dimer 8

Figure S9. Average of all pH titration curves 9

Table S1. Characterization of all PSII proteins regarding gene name, transmembrane helices and co-factors 10

References 11

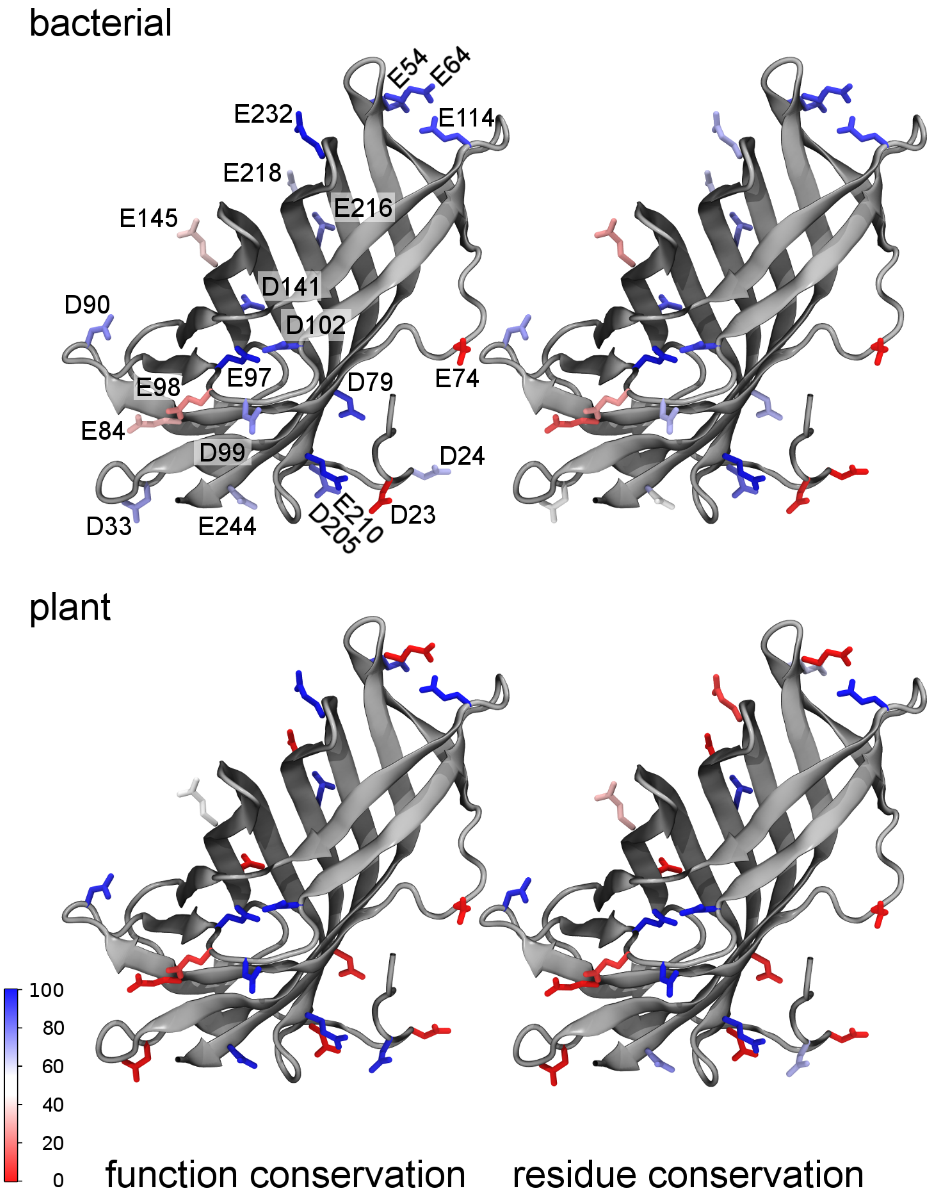

Figure S1. Conservation pattern of PsbO surface carboxylates in plants and bacteria. Conservation of carboxylates on the surface of PsbO in bacterial(top) and plant(bottom) sequences shown from high(blue) to low(red). Comparison of conservation of function (on the left) and conservation of the residue (on the right).

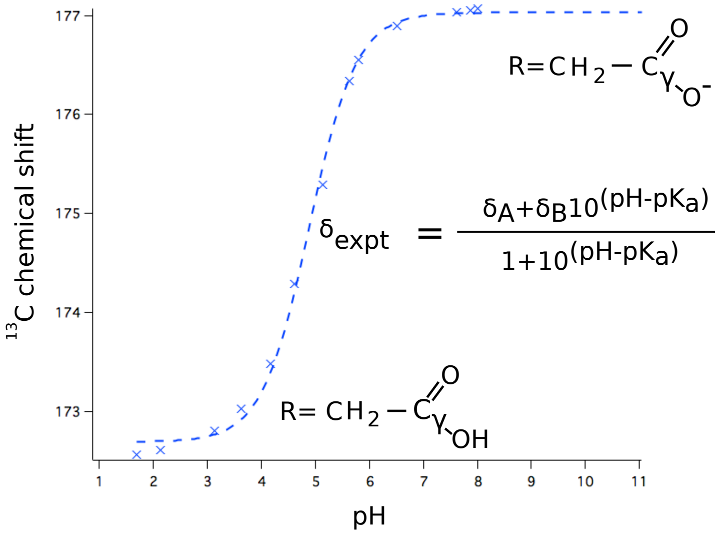

Figure S2. Exemplary pH titration curve. pH titration curve of Asp141(134) with Henderson-Hasselbalch fit function.

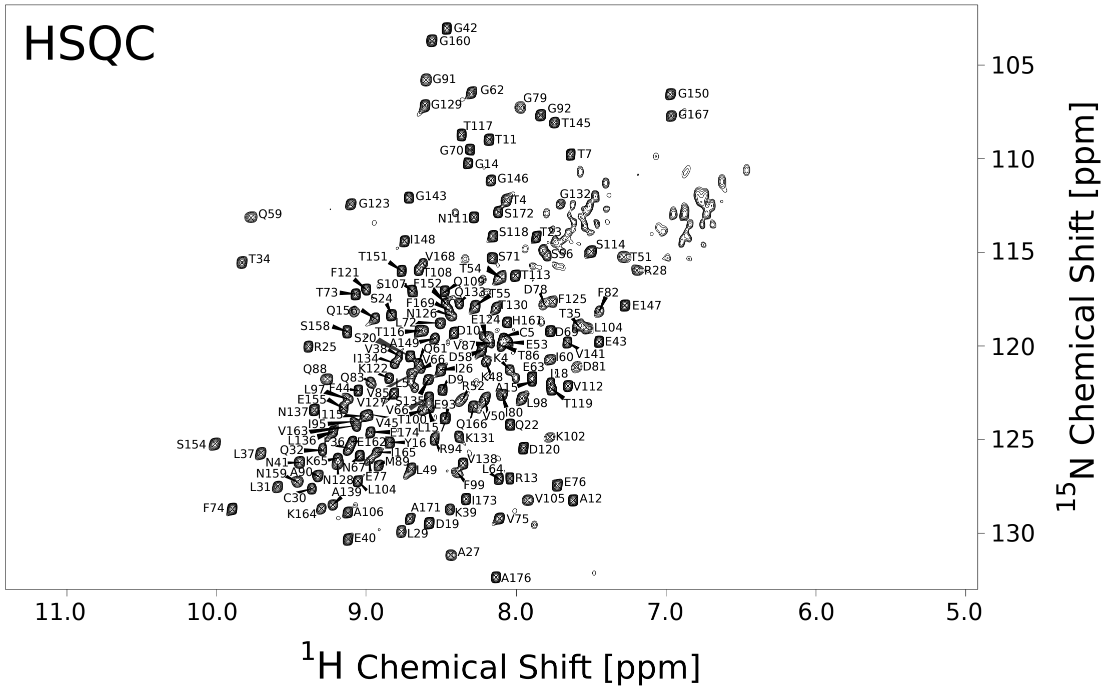

Figure S3. ^{15}N-^{1}H HSQC spectrum of PsbO. All obtained resonance assignments of amide group signals are indicated by the respective residues in one letter code in a ^{15}N-^{1}H HSQC spectrum of PsbO recorded at pH 6.5.

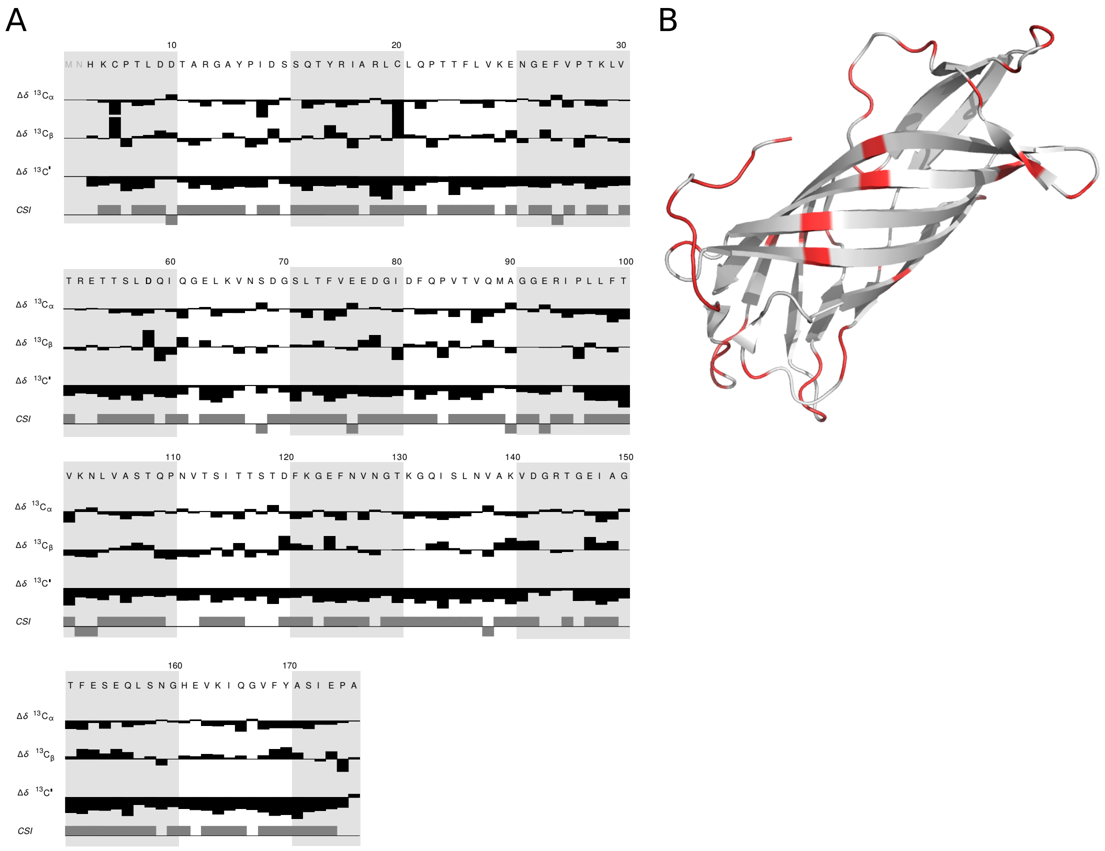

Figure S4. Comparison of chemical shift index and crystal structure. A The protein backbone chemical shifts are used for a secondary structure analysis of PsbO. The first three rows show the differences between observed Cα, Cβ and CO chemical shifts and average random coil chemical shifts from the BMRB[1] for each residue. The chemical shift index (CSI) for each residue of PsbO is shown in the bottom row. Positive and negative CSI values indicate α-helical and β-sheet secondary structures, respectively, and random coil segments are characterized by a CSI close to 0. **B** The structure of PsbO (PDB file 5G39[2]) is shown in cartoon representation, with residues highlighted in red for which the CSI differs from the secondary structure motif observed in the crystal structure.

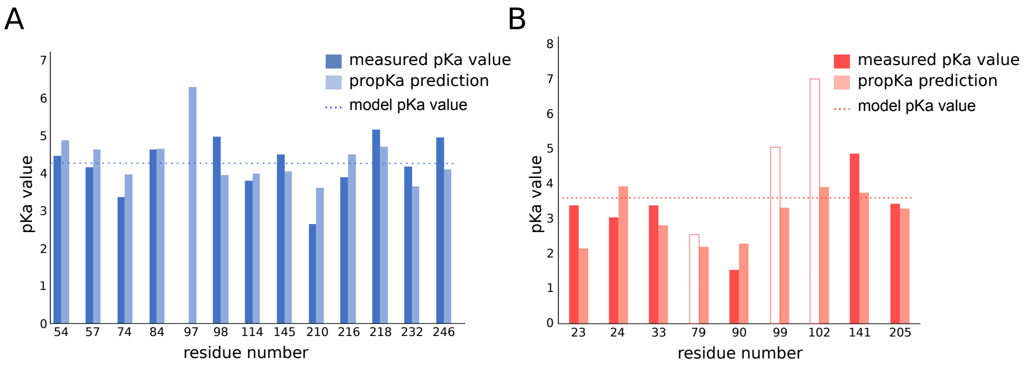

Figure S5. Comparison of pK_a values with ProPka[3] prediction program. A The measured pK_a values of carboxyl groups of glutamic acid residues are shown in deep blue, and the ProPka data in light blue. The dotted line indicates the model pK_a value of glutamic acid[4] in solution. **B** The measured pK_a values of carboxyl groups of aspartic acid residues are shown in deep red, and the ProPka data in light red. White bars represent residues with minimal and maximal pK_a values. The dotted line indicates the model pK_a value of aspartic acid[4] in solution.

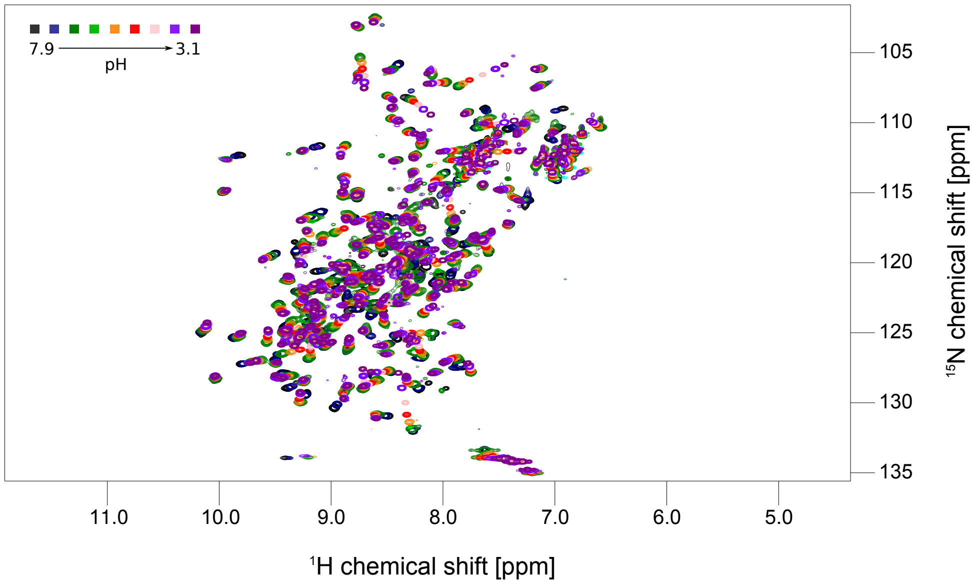

Figure S6. ^{15}N-^{1}H HSQC titration spectra. Overlay of ^{15}N-^{1}H HSQC spectra of PsbO, recorded at different pH values ranging from 7.9 (dark blue) to 3.1 (purple).

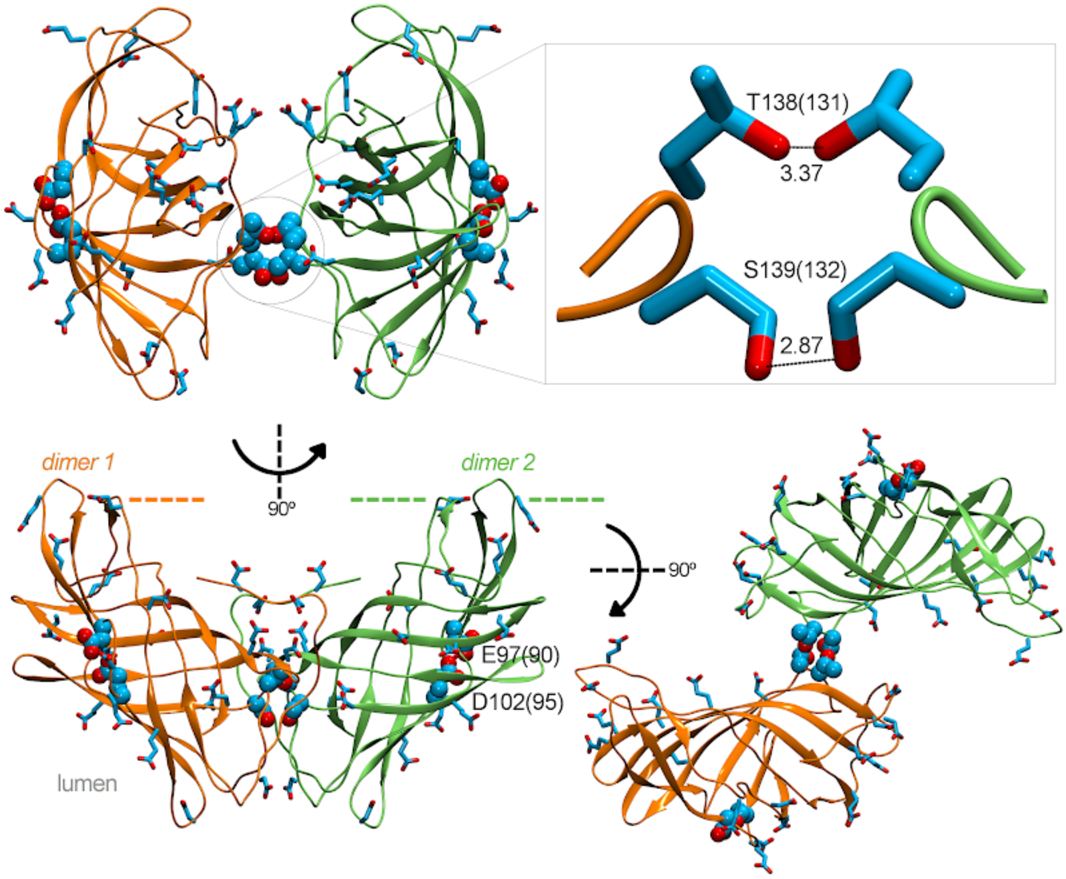

Figure S7. PsbO-mediated interactions between Photosystem II (PSII) dimers. The crystal structure PDB ID:4PJ0 of the PSII dimer[5] and the crystal structure PDB ID:5G38 of PsbO-β[2] were used to generate a structural model of the PsbO-mediated interactions between PSII dimers. The PsbO subunits (orange and green) of two neighboring PSII dimers form an interface based on hydrogen-bonding between T138 and S139.

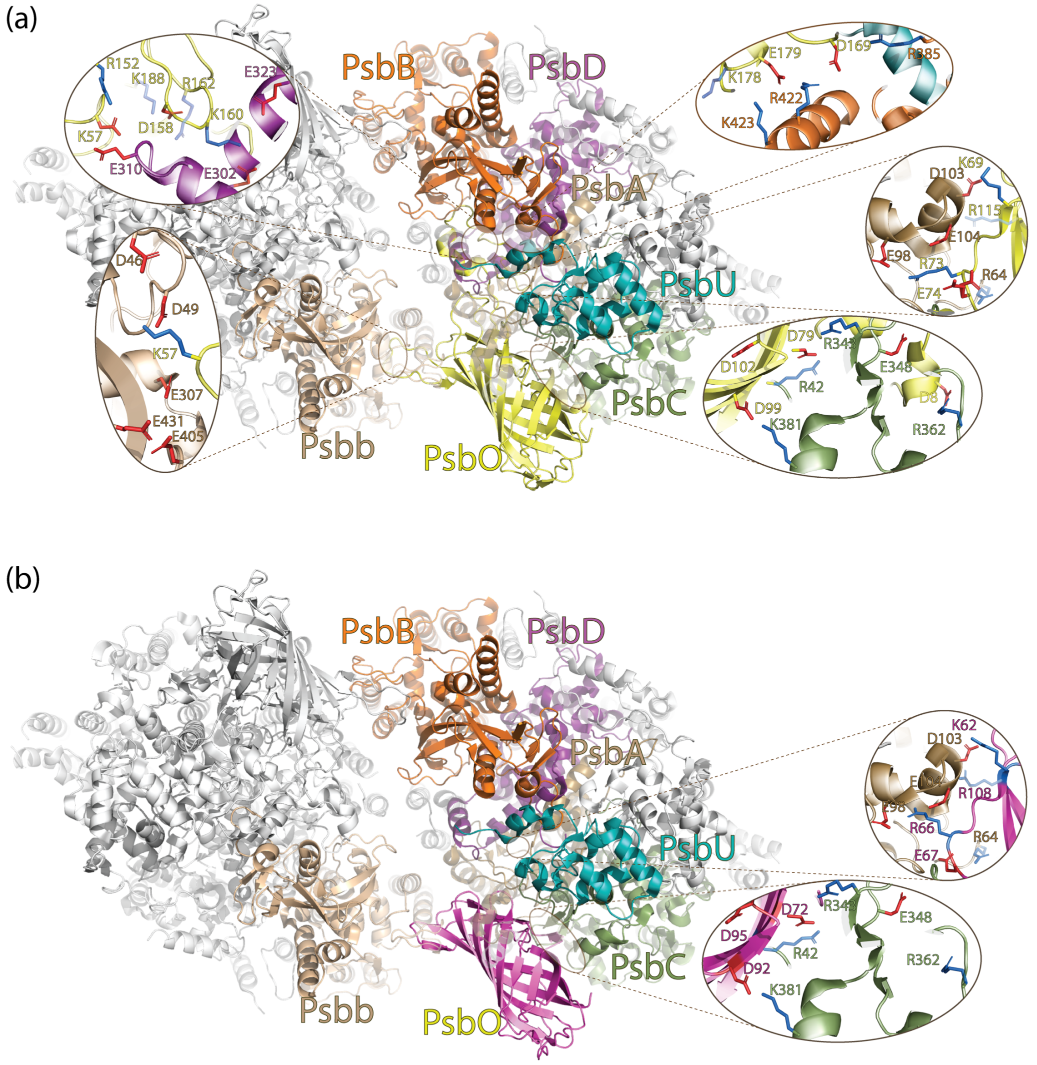

Figure S8. Interactions of PsbO with different subunits in a PSII dimer. The PsbO subunits establish contacts to other subunits within a PSII dimer (PDB 5b5e[6]). **A** Full-length PsbO interacts with Psbb, PsbB, PsbC, PsbA and PsbU. **B** The β-barrel construct of PsbO binds to PsbC and PsbA.

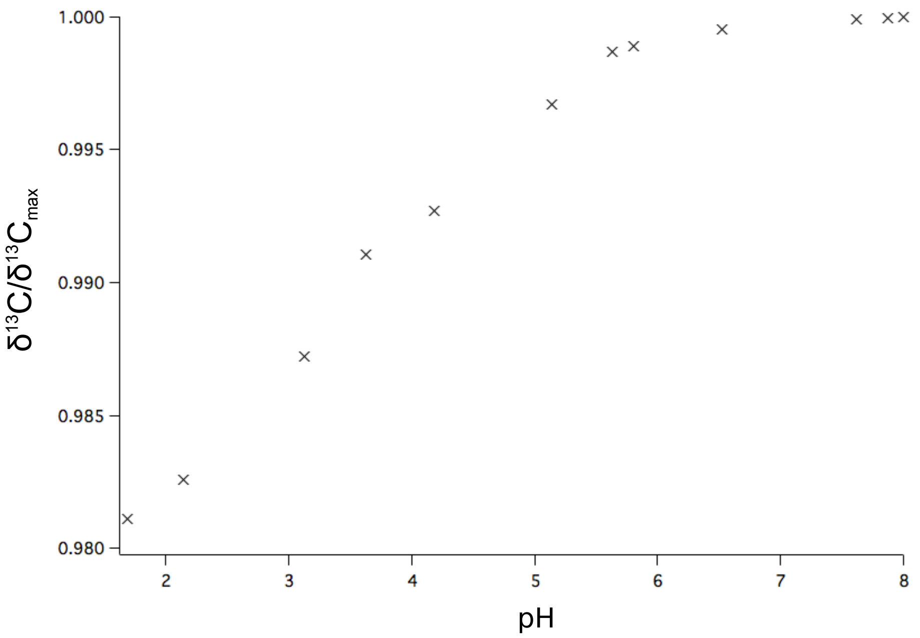

Figure S9. Average of all pH titration curves. The average of all aspartate and glutamate pH titration curves, normalized to the alkaline plateau level, indicates an elongated transition to the protonated state of PsbO and an inflection point around pH 4.1. For each residue we calculated a normalized curve, where we divided the individual ^{13}C ppm values by the maximal ^{13}C ppm value at pH 8. We added the normalized curves for each pH value individually and divided the values by the number of curves added.

Table S1. Characterization of all PSII proteins regarding gene name, transmembrane helices and co-factors. The gene and protein names of the different PSII subunits are shown in the first two columns[7]. The number of transmembrane helices(TMH) and chlorophyll molecules per photosystem II is given in the following four columns both for cyanobacteria and plants; the types of additional co-factors are given in the last column.

Gene	Subunit	TMH		Chlorophylls		Co-factors
		Cyano	Plants	Cyano	Plants	
psbA	A (D1)	5	5	3	3	Y_Z, Pheo a, Q_B
psbB	B (CP47)	6	6	16	16	—
psbC	C (CP43)	6	6	14	14	—
psbD	D (D2)	5	5	3	3	Y_D, Q_A
psbE	E	1	1	no	no	Cyt b559 heme
psbF	F	1	1	no	no	Cyt b559 heme
psbH	H	1	1	no	no	—
psbI	I	1	1	no	no	—
psbJ	J	1	1	no	no	—
psbK	K	1	1	no	no	—
psbL	L	1	1	no	no	—
psbM	M	1	1	no	no	—
psbO	O	0	0			—
psbP	P	—	0			—
psbQ	Q	—	0			—
psbR	R	—	1			
psbS	S	—	4			—
psbTc	T	1	1			—
psbTn	—	—	0			—
psbU	U	0	—			—
psbW	W	—				—
psbV	V	0	—			Cyt c550 heme
psbX	X	1	1			—
psbY	Y	1	1			—
psbZ	Z	2	2			—
Ycf12	—	1	—			—

References

[1] Ulrich, E. L., Akutsu, H., Doreleijers, J. F., Harano, Y., Ioannidis, Y. E., Lin, J., Livny, M., Mading, S., Maziuk, D., Miller, Z., Nakatani, E., Schulte, C. F., Tolmie, D. E., Kent Wenger, R., Yao, H., and Markley, J. L. *Nucleic Acids Res.* **2008,** *36*, 402–408.

[2] M. Bommer, A. N. Bondar, A. Zouni, H. Dobbek, H. Dau, *Biochemistry* **2016**, DOI 10.1021/acs.biochem.6b00441

[3] M. H. M. Olsson, C. R. SØndergaard, M. Rostkowski, J. H. Jensen, *J. Chem. Theory Comput.* **2011**, *7*, 525–537.

[4] R. L. Thurlkill, G. R. Grimsley, J. M. Scholtz, C. N. Pace, *Protein Sci.* **2006**, *15*, 1214–1218.

[5] J. Hellmich, M. Bommer, A. Burkhardt, M. Ibrahim, J. Kern, A. Meents, F. Müh, H. Dobbek, A. Zouni, *Structure* **2014**, *22*, 1607–1615.

[6] Tanaka, A., Fukushima, Y., and Kamiya, N. T. *J. Am. Chem. Soc.* **2017,** *139*, 1718–1721.

[7] Berman, H., Westbrook, J., Yukang, F., Gilliland, G., Bhat, T., Weissig, H., Shindyalov, I., and Bourne, P. *Nucleic Acids Res.* **2000,** *28*, 235–242.
